# Effect of Growth Regulators on *In Vitro* Micropropagation of Potato (*Solanum tuberosum* L.) Gudiene and Belete Varieties from Ethiopia

**DOI:** 10.1155/2021/5928769

**Published:** 2021-02-08

**Authors:** Sunil Tulshiram Hajare, Nitin Mahendra Chauhan, Girum Kassa

**Affiliations:** Department of Biology, College of Natural and Computational Sciences, Dilla University, Dilla 419, Ethiopia

## Abstract

**Aim:**

Potato (*Solanum tuberosum* L.) is one of the important crops in Ethiopia which has a crucial role in nutritional security, poverty alleviation, and income generation. The aim of the present investigation is to develop an efficient *in vitro* propagation protocol for Belete and Gudiene potato varieties by using lateral bud as explants.

**Materials and Methods:**

Shoot initiation was achieved by inoculating buds on full-strength MS Murashige and Skoog medium (MS) fortified with variable concentrations of BAP and NAA. Basal MS was used as control throughout the experiment.

**Results:**

Results of our study showed that best shoot initiation was obtained on MS medium supplemented with 1.5 mg/l BAP + 3.0 mg/l NAA for Gudiene variety, whereas 1.0 mg/l BAP and 2.0 mg/l NAA produced more shoots in Belete variety. The initiated shoots increased two- to three-fold upon subculture on the MS medium fortified with varying concentrations of BAP and Kinetin. The highest numbers of multiple shoots were obtained in the MS medium containing 2.5 mg/l Kinetin. The combined effect of BAP and Kinetin did not produce any additional positive effect for shoot multiplication. Rooting percentage and number of roots/shoot were found best on the MS medium fortified with 1.0 mg/l IBA + 0.5 IAA.

**Conclusions:**

The variety Gudiene was found best for shoot initiation and root formation, while Belete variety proved its superiority for multiple shoot formation. A total number of 82.66% of plantlets were acclimatized under field conditions. This work indicates the practical applicability of plant tissue culture using lateral bud as explants is effective for micropropagation of potato *in vitro*.

## 1. Introduction

Potato (*Solanum tuberosum* L.) is one of the most important crops in Ethiopia which has a crucial role in nutritional security, poverty alleviation, and income generation [1]. Due to increasing urbanization, the potato used as processed products such as French fries and crisps is gaining popularity [2]. Compared to other African countries, Ethiopia has great potential for potato production due to favorable climatic conditions. About 70% of land comes under highland areas which are suitable for potato cultivars. Since the highlands are also home to almost 90% of Ethiopia's population, potato could play a key role in ensuring national food security [3].

Potato seed production in Ethiopia is informal, which in most cases operates by recycling planting materials from previous crop harvests. At present, 98.7% of the requirements for potato seed are met by informal seed supply. Sources of informal seed vary and include seeds from neighboring farmers, friends, relatives, merchants, and markets where potato is sold for consumption. Shortage of seed potato has been recognized as one of the most important factors limiting potato production in developing countries [4].

The main problem of growing potatoes worldwide is economic losses due to late blight, which is caused by *Phytophthora infestans* which can destroy potato plants within two weeks in wet conditions. Blight can survive even under adverse conditions. The pathogen, however, invades and infects potatoes in the field via zoosporangia, which disperse via soil, water, rain splash, and wind. Due to progressive accommodation of viral disease in seed stock, availability of good-quality seed is a major constraint in potato production, which is approximately 50% of the total production cost. Besides high cost of seed, potato propagation is also characterized by low multiplication rate of only 4–6 times [5].

Shortage of good-quality seeds has been recognized as the single most important fact of limiting potato production in the developing countries. Fortunately, potato has been an early beneficiary of advances in conventional and modern biotechnology, resulting in their use for solving practical problems relating to potato cultivation and improvement. Meristem culture was possibly the first biotechnological approach used to eliminate viruses from systemically infected potato clones. Over the years, this technique has been successfully combined with micropropagation to produce disease-free potato seeds. Rapid multiplication of these disease-free clones using micropropagation coupled with conventional multiplication methods has now become an integral part of seed production in many countries [6].

The most economically successful tissue culture technique is the alternative means of plant vegetative propagation known as micropropagation. The significant advantage offered by micropropagation over conventional methods is that, in a relatively short time and space, a large number of plants can be produced from a single individual independent of the seasons [7]. Micropropagation is essential for highly heterozygous species such as potato for producing uniform plants [8]. Potatoes can be micropropagated rapidly on a large scale by meristem and shoot-tip cultures, proliferation by axillary shoots developed from in *vitro*-cultured nodal cuttings [9–11], and production of adventitious shoots directly on explants or indirectly via a callus phase. The current advances are seen as the beginning of the second “Green” in agriculture and are expected to make farming more efficient, profitable, and environmentally safe [5].

Considering the abovementioned prelude, the aim of the present investigation is to develop an efficient *in vitro* propagation protocol for Belete and Gudiene potato varieties from lateral bud as explants in potato (*Solanum tuberosum* L.).

## 2. Materials and Methods

### 2.1. Study Area

The present investigation was carried out at the Biotechnology Laboratory of Areka Agricultural Research Center (AARC), Southern Nations Nationalities and People's Regional State (SNNPRS), Wolaita Zone, Ethiopia. The centre is 300 km away from the capital city of Ethiopia.

### 2.2. Explant Sterilization

Two popular potato varieties, Gudiene and Belete, were collected from the Areka Agricultural Research Center, Areka, Ethiopia, and grown under green house conditions to obtain buds as explants after 4-5 weeks [12]. The geographic extent of Areka is 7°3′26″ to 7°4′24″ northern latitude and from 37°40′52″ to 37°41′30″ eastern longitude. Throughout the study, fresh disease-free lateral buds about 2.0 to 2.5 cm long were taken after 4 weeks of incubation in green house. The explants immediately after taken from the mother plant were washed by tap water and double distilled water. After primary sterilization, the explants were treated with 70% ethanol for 90 seconds and immediately washed with distilled water. Next, the explants were dipped in 1% HgCl_2_ (mercuric chloride) for 4 to 8 minutes [5]. After this two-step sterilization procedure, the sterilized explants were transferred to laminar air flow cabinet. Finally, they were washed 4 to 5 times with sterilized distilled water to avoid contamination.

### 2.3. Shoot Initiation

A full-strength MS medium has been used throughout experiments [12–14]. Four variable concentrations of BAP (0, 0.5, 1, 1.5, and 2.0 mg/l) combined with different combinations (0, 1, 2, 3, and 4 mg/l) of NAA were tested for shoot initiation. Each treatment composed of 3 shoots/vessel with three replications. The culture was placed on the growth room chamber with 16 hours photoperiod (8 hours dark) and 2700 lux light intensity at 25 ± 2°C [15]. The white fluorescent lights were 28 cm away from the top of the culture vessels. The stock solution composition, agar concentration, and other physical conditions were the same for all the treatments. After a month of growth, every change in growth was carefully observed and recorded. A plant growth regulator-free medium was used as a control.

### 2.4. Shoot Multiplications

After 30 days of growth on the culture initiation medium, young and healthy microshoots were cultured on a shoot multiplication, full-strength MS basal medium [12, 13], containing different concentrations of BAP (1, 1.5, 2, 2.5 mg/l) and KN (1, 1.5, 2, 2.5 mg/l) individually or in combination (1.5 BAP + 2 KIN, 2 BAP with 1.5 KIN). Five shoots were inoculated per jar with three replications for each treatment. The shoots after 2 weeks were aseptically taken out and inoculated on the multiplication medium. The culture vessels were properly sealed, labeled, and randomly placed on the growth room chambers with the same culture conditions (temperature, photoperiod, and light intensity) as from the beginning of experiment. The control medium was the basal MS medium. The development of shoots at different stages of multiplication was observed and recorded at specific intervals.

### 2.5. Rooting

The developed shoots were aseptically removed from culture vessel and, after washing, inoculated on a full-length MS medium fortified with variable concentrations of auxins, IBA (0.5, 1, 1.5, 2 mg/l) and IAA (0.5, 1, 1.5, 2 mg/l) alone, as well as in combinations (0.5 IBA with 1 IAA, 1 IBA with 0.5 IAA). The PGR-less MS medium (basal) was used as a control. The number of shoots per culture vessel was five with three replications for each treatment [5].

## 3. Results

### 3.1. *In Vitro* Shoot Induction and Viability of Explants

**Figure 1:**
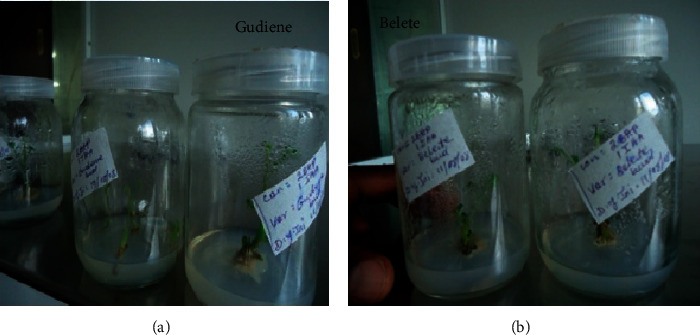
*In vitro* shoot initiation in Gudiene and Belete varieties of potato.

Potato shoot cultures were established from lateral bud segments of *Solanum tuberosum* L. Gudiene and Belete varieties. Shoots started to initiate within a week of inoculation. The buds started to form shoots. However, the mean number of shoots did not produce statistically different results; both Gudiene and Belete varieties showed mediocre response for shoot induction at different concentrations of auxins and cytokinins. The combination of 1.5 mg/l BAP and 3.0 mg/l NAA proved best in Gudiene, whereas 1.0 mg/l BAP and 2.0 mg/l NAA produced more shoots in Belete. The rest of the treatments failed to produce statistically significant values, which proved that the combinations of 1.5 mg/l BAP and 3.0 mg/l NAA and 1.0 mg/l BAP and 2.0 mg/l NAA are the best to shoot initiation in Gudiene and Belete, respectively, in this investigation. Simultaneously with shoot initiation, some root development was also observed above the medium surface after 4 weeks of inoculation. This may be due to fortification of NAA (Table 1; Figure 1).

### 3.2. The Effect of Cytokinin Type and Concentration on *In Vitro* Shoot Multiplication

**Figure 2:**
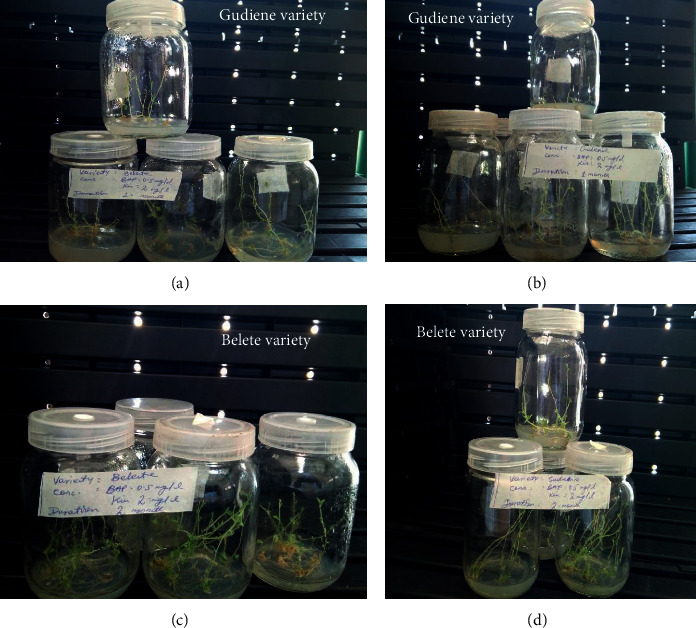
Shoot multiplication in Gudiene and Belete varieties of potato.

The lateral bud segments showed multiplication after two weeks of culture in media supplemented with all concentrations of BAP and kinetin. Although variations were observed in the response of explants to plant growth regulator treatment at the earlier subculture, the results reported here reflect the status of multiplication after three subcultures, reported on the performance per shoot basis and not as cumulative output after three subcultures (Table 2; Figure 2). Individual as well as combined influence of BAP and Kinetin produced significant results for multiple shoot formation in both varieties of potato at all concentrations tested. Multiple shoot formation/explants had been recorded, and the mean numbers of shoots were varied among the treatments (Table 2).

All the treatments tested produced considerably multiple shoots (3.71–8.33 shoots/explants) on the MS medium fortified with individual BAP and Kinetin, as well as in combination (Table 2). The variety Gudiene produced the highest shoots on the MS medium fortified with 1.0 mg/l BAP, whereas the MS medium supplemented with equal concentration of BAP and Kinetin (2.5 mg/l) produced shoots with the highest length (7.64 cm). Increasing trend in multiple shoot formation had been observed in the variety Belete, and the treatment (MS + 2.5 mg/l Kinetin) which failed to produce multiple shoots in Gudiene was found effective. The MS medium incorporated with 2.0 and 2.5 mg/l Kinetin was found best as it produced 8.33 and 8.35 shoots/explant with 7.33 and 7.36 cm shoot length, respectively. This may be due to the difference in the genomic makeup of the two varieties. In overall shoot formation, the variety Belete proved its superiority over Gudiene (Table 2; Figure 2).

### 3.3. The Effect of IBA and IAA on *In Vitro* Root Formation

**Figure 3:**
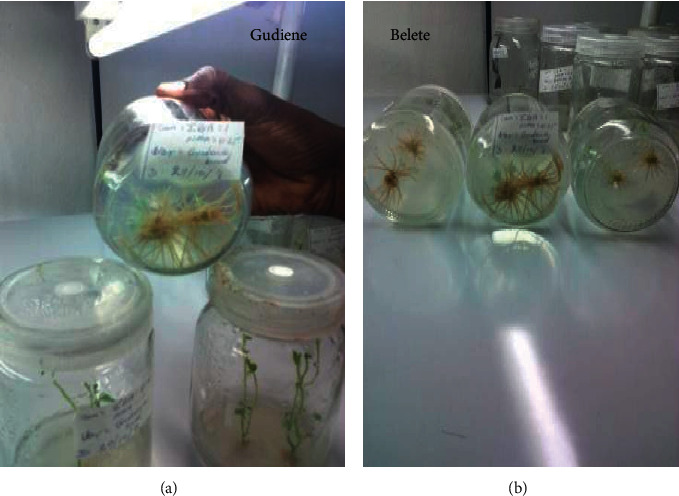
*In vitro* root formation in Gudiene and Belete varieties of potato.

The well-grown shoots of about 1 to 1.5 cm in height were selected and inoculated on a full-strength MS medium fortified with variable concentrations of IBA and IAA individually as well as in combination. Both individual and the combination of IBA and IAA produced significant results for root formation in both potato genotypes at all concentrations tested. The frequency of roots/shoots and the length of roots/shoot observed significantly varied among all the treatments used (Table 3; Figure 3). The MS medium supplemented with 1 mg/l IBA + 0.5 mg/l IAA produced significantly more number of roots/shoot and root length/shoot in both varieties (Table 3; Figure 3). For the rooting, both the genotypes produced profound roots on the medium containing 1 mg/l IBA and 0.5 mg/l IAA. The cultivar Gudiene was found better than Belete. The individual effect of IBA and IAA was not promising for rooting.

## 4. Discussion

Potatoes can either be propagated sexually by seeds or asexually by means of tubers. Seed potato tubers are mainly utilized for multiplication and production [16]. This methodology has a number of disadvantages such as low rate of multiplication and vulnerable risk to various diseases. The plant tissue culture technique has become an alternative and gained popularity as an alternative method for vegetative propagation of plants in recent years. As a modern technology, the plant tissue culture has a great potential to meet the ever-increasing world demand. It has made significant contributions for the betterment of agricultural sciences in recent times, and today, it contributes an indispensable tool in modern agriculture. Because of tissue culture, today we are able to micropropagate a large number of plants from a single seed or explants with select desirable traits; reduce the amount of space required for field trials; and generate disease-free plants through careful selection and sterile techniques.

Shoot initiation and multiplication was a long-lasting problem of potato tissue culture. Shoots were produced sporadically, but a simple and consistent regeneration procedure was missing. Apparently, potato callus cannot be manipulated to regenerate shoots as easily as in other plant species. The results of this study are in line with the findings of Jarret et al. [17] and Kikuta and Okazawa [18] who found the best shoot initiation on the MS medium supplemented with BAP and NAA at the concentration ranged between 1.5–3.0 mg/l BAP and NAA. High concentration of BAP to NAA more than 3.0 mg/l and lower than 1.0 mg/l BAP and NAA completely failed to initiate shoots in Gudiene and Belete. According to Berrie [19], synthetic cytokinins are inhibitory to shoot growth at high concentrations. Different studies suggested the combination of growth hormones varies from potato varieties as well on the type of explants used. Likewise, Kong et al. [20] used apical meristems of potato as explants to initiate *in vitro* cultures on a modified MS solid medium supplemented with BAP, NAA, and GA3. Rout et al. [21] found that BAP, kinetin, and ascorbic acid give the best results for regeneration of multiple shoot formation from apical shoots in potato. Shah et al. [22] found that the highest stem length and the largest single node in potato can be obtained in an MS medium supplemented with 0.5 mg/l NAA. The effect of different concentrations of GA3 and BAP on *in vitro* multiplication of the potato variety Desiree was studied by Asma et al. [23], and they found that the maximum shoot length was obtained at 4 mg/l GA3. Maximum number of shoots was obtained when 2 mg/l BAP was used. A similar type of research on maximum number of shoot induction on 2 mg/l of BAP was also reported in the PPR-1 mulberry variety [24]. Thus, it is clear from the abovementioned studies that standardization of protocol varies on the basis of explants used and potato variety. The result of the proposed study demonstrates that best shoot initiation was obtained on an MS medium supplemented with 1.5 mg/l BAP + 3.0 mg/l NAA for the Gudiene variety, whereas 1.0 mg/l BAP and 2.0 mg/l NAA produced more shoots in the Belete variety.

The techniques of *in vitro* shoot multiplication have been successfully used in potato biotechnology for nearly 60 years. Some of these techniques such as culture of callus, roots, and shoots are considered mature, well-elaborated, routine techniques in which no major improvements have been implemented for a number of years. In the present investigation, an attempt has been made to develop rapid multiple shoot formation from shoot tips of buds by using BAP and Kinetin [25–30]. The results of this study can be discussed with those Esna-Ashari and Villiers [31] who recorded multiple shoots from potato buds from an MS medium supplemented with BAP, whereas Mix and Sixin [32] failed to achieve multiple shoots with cytokinins. This may be due to the genotype difference in the variety tested. According to many earlier reports, the combined effect of BAP and Kinetin was better for shoot multiplication in the number of crops [17, 33, 34].

The results of the present study are in agreement with the findings of Bajaj and Dionne [35] who improved the growth of excised potato roots by using IBA and IAA with 1.0 and 0.5 mg/l. The results also largely support the findings of Rabbani et al. [23] who reported the best rooting response in potato when IBA concentration was at higher proportion than NAA and IAA in a combination of the two. Another research established that the use of 2.5 mg/l of IBA is good to improve root initiation of potato plantlets which is at par with the present finding that rooting increases with high IBA concentration in a combination of IAA or NAA [36]. However, these earlier reports involved different cultivars with different physiological conditions and may be due to that the findings of Chapman [37] are not in match with the present study which could not establish the rooting with the IBA and IAA. A similar type of higher number of root induction on combination of IAA and IBA was achieved in *Mirabillis jalapa* [38] and *Andrographis echioides* [39].

## 5. Conclusions

In the end, we conclude that an effective protocol for the micropropagation of two potato varieties from Ethiopia was optimized. It was observed that best shoot initiation was obtained on the MS medium supplemented with 1.5 mg/l BAP + 3.0 mg/l NAA for the Gudiene variety, whereas 1.0 mg/l BAP and 2.0 mg/l NAA produced more shoots in the Belete variety. On the other hand, the MS medium containing 1 mg/l IBA and 0.5 mg/l IAA was found to be effective for root generation in both the varieties studied. A total of 82.66% of plantlets was acclimatized under field conditions. As such, this protocol will provide the base for the mass production of studied varieties through *in vitro* techniques. In the end, this work indicates the practical applicability of plant tissue culture using lateral bud as an explant is effective for micropropagation of potato *in vitro*.

## Figures and Tables

**Table 1:** The effect of BAP and NAA on shoot initiation in Gudiene and Belete varieties of potato.

Treatments (MS + BAP + NAA (mg/l)	No. of explants inoculated	No. of shoots initiated	Unviable explants	Shoot initiation percentage
Variety- Gudiene				
Basal medium (control)	9	3.40	5.66	37.77^cdef^
0.5 + 1.0	9	5.68	3.32	63.11^cd^
1.0 + 2.0	9	6.25	2.75	69.44^c^
1.5 + 3.0	9	8.95	0.05	99.44^a^
2.0 + 4.0	9	5.55	3.45	61.66^cde^

Variety- Belete				
Basal medium (control)	9	4.10	4.90	45.55^cde^
0.5 + 1.0	9	5.61	3.39	62.33^cd^
1.0 + 2.0	9	8.81	0.19	97.88^a^
1.5 + 3.0	9	7.10	1.90	78.88^b^
2.0 + 4.0	9	5.95	3.05	66.11^c^
LSD				**14.26**
CV (%)				**10.56**

^*∗*^Means represented by the same lowercase are not significantly different, whereas those with different letters are significantly different.

**Table 2:** The effect of various concentrations of BAP and Kinetin on *in vitro* shoot multiplication in potato Gudiene and Belete varieties.

Variety	Growth regulators (mg/l)		Mean no. of nodes/explants	No. of shoots/explants	Mean no. of shoot length (cm)
	BAP	Kin			
Gudiene	0	0	00	00	00
	1.0	1.0	6.40	7.77	7.15
	1.5	1.5	5.41	4.60	5.31
	2.0	2.0	4.29	3.71	4.21
	2.5	2.5	7.72	8.11	7.64
	1.0	Absent	**8.33**	**8.33**	**5.50**
	1.5		4.67	6.00	4.50
	2.0		3.67	5.33	4.08
	2.5		3.70	5.35	4.10
	Absent	1.0	5.67	5.00	4.67
		1.5	6.33	6.00	4.67
		2.0	7.00	6.67	5.83
		2.5	6.31	6.02	4.65

Belete	0	0	00	00	00
	1.0	1.0	6.67	7.00	6.83
	1.5	1.5	4.67	4.67	5.17
	2.0	2.0	**3.67**	**4.00**	4.50
	2.5	2.5	4.60	4.61	5.14
	1.0	Absent	7.3	6.67	5.00
	1.5		5.00	5.33	4.33
	2.0		5.33	5.00	4.17
	2.5		5.02	5.35	4.30
	Absent	1.0	6.33	5.67	4.67
		1.5	6.67	7.00	5.33
		2.0	7.67	8.33	7.33
		2.5	**7.68**	**8.35**	**7.36**
LSD					
	**1.02**		**1.02**	**0.83**	
CV (%)					
	**10.96**		**10.99**	**10.90**	

^*∗*^Values represent the mean of three replications.

**Table 3:** The effect of various concentrations of IBA and IAA on *in vitro* shoot multiplication in Gudiene and Belete varieties of potato.

Variety	Growth regulators (mg/l)		Mean no. of roots/shoots	Mean no. of shoot length (cm)
	IBA	IAA		
Gudiene	0	0	1.29	1.55
	0.5	1.0	8.67	3.83
	1.0	0.5	**21.12**	**7.87**
	0.5	Absent	9.30	3.91
	1.0		15.54	5.41
	1.5		11.21	6.58
	2.0		12.32	6.61
	Absent	0.5	9.32	3.95
		1.0	**15.51**	**5.44**
		1.5	11.21	6.60
		2.0	12.30	6.61

Belete	0	0	1.27	1.56
	0.5	1.0	8.68	3.83
	1.0	0.5	18.00	7.84
	0.5	Absent	9.28	3.90
	1.0		15.56	5.43
	1.5		11.21	6.55
	2.0		12.30	6.61
	Absent	0.5	9.35	3.94
		1.0	15.49	5.44
		1.5	11.20	6.60
		2.0	12.32	6.64
LSD				
		**1.03**		**2.97**
CV (%)				
		**6.20**		**8.14**

^*∗*^Values represent the mean of three replications.

## Data Availability

Raw data were generated at the Areka Tissue Culture laboratory. Derived data that supported the findings of this study are available from the corrosponding author (Hajare ST) on request.
